# Editorial for the Special Issue, “Artificial Intelligence Applications in Cancer and Other Diseases”

**DOI:** 10.3390/biomedicines14020278

**Published:** 2026-01-27

**Authors:** Raji Sundararajan

**Affiliations:** School of Engineering Technology, Purdue University, West Lafayette, IN 47907, USA; raji@purdue.edu; Tel.: +1-765-494-6912

Advances in medical sciences can mean enhanced quality of life at affordable prices. With over 50% of bankruptcies in the USA being medical-related, it is critical that emerging artificial intelligence (AI) technology and related products be used to enhance the wellness and welfare of ordinary citizens at affordable rates. Working toward this goal, this Special Issue, “Artificial Intelligence Applications in Cancer and Other Diseases”, features eight research articles and one systematic review. The topics of these articles include auto machine learning (ML) and convolutional neural networks (CNNs) in diabetes mellitus research; deep-learning-based real-time organ localization and transit time estimation in wireless capsule endoscopy; deep-learning-based surgical treatment recommendations; the use of machine learning models in obesity to predict weight loss after bariatric surgery; prostate cancer detection using an equilibrium optimization algorithm with deep learning (DL); a radiotherapy dose map-guided deep learning method for predicting pathological complete response in esophageal cancer; effective invasiveness recognition in imbalanced data using semi-automated segmentations of lung nodules; ASNET, a novel AI framework for accurate ankylosing spondylitis diagnosis based on MRI; and the use of machine learning models in sepsis outcome prediction for ICU patients (a systematic review).

The novelty of the first article on diabetes lies in the use of auto machine learning and CNNs to study an induced-diabetes animal model. Using female rats, the authors studied the impact of diabetes on female reproductive organs, namely, the vagina, ovaries, and uterus. The experimental results indicated that uterine tissue can be diagnosed with an accuracy of 95.5% and 85.8%, respectively, for the two different compounds used. This research underscores the efficacy of classification with two AI algorithms, CNN and ML. The authors of the second article studied wireless capsule endoscopy (WCE) videos of 72 patients using deep learning. The results indicated high performance in organ classification, achieving an accuracy, sensitivity, and specificity of >95% for each organ (the stomach, small intestine, and colon), with an overall accuracy and F1-score of 97.1%. These findings suggest that the use of WCE enhances diagnostic accuracy and efficiency, making it a valuable tool in clinical practice for diagnosing and managing GI diseases. The third article proposes the use of recent deep learning techniques in surgical treatment recommendations and nonsurgical prognosis status classification. The fourth article indicates that in severe obesity, ML models could be used to assist in the selection of patients for bariatric surgery.

The authors of the fifth article used deep learning to enable prostate cancer detection with MRI images. They propose an equilibrium optimization algorithm to detect and classify prostate cancer, the most common cancer in men worldwide. With about 1.5 million new cases annually (worldwide in 2025) and over 313,000 cases and 35,770 deaths in the US, the use of AI and its subsets ML and DL represents hope for patients. However, they have to be affordable. The sixth contribution is another article on deep learning, specifically, its use in predicting pathological complete response (ypCR) in esophageal cancer patients after neoadjuvant chemotherapy followed by surgery. It highlights the potential of dose-guided DL in ypCR prediction, necessitating larger, multicenter studies to validate the results. The seventh article deals with invasiveness, an important factor in expected survival rates. Using semi-automated segmentations of lung nodules, the authors demonstrate a specificity improvement of 14.3%. This method is more useful with imbalance data. The eighth and last research article is on ankylosing spondylitis (AS), a chronic, painful, progressive disease usually seen in the spine. Using CNNs, deep features were generated based on MRIs. These feature selectors were employed with k-nearest neighbors, and good classification performance was reported. The ability to diagnose AS using only axial images, without the need for contrast-enhanced MRI, represents both a significant advancement in healthcare and reduced treatment costs.

The systematic review article explores 19 articles on sepsis, published from 2014 to 2024, finding that through the real-time integration of routine laboratory and clinical data, ML-based tools, such as logistic regression, random forests, and neural networks, can assist in clinical decision-making in sepsis and enhance the consistency and quality of its management across various healthcare units, including ICUs with limited resources.

To summarize, these nine articles demonstrate effective clinical applications of AI and its subsets, ML, NN, and DL, showing that they are valuable tools with the potential to advance the medical field and that their clinical applications could be beneficial to patients by allowing lower costs and better treatments.

